# Levels of neutralizing antibodies against resident farm strain or vaccine strain are not indicators of protection against PRRSV-1 vertical transmission under farm conditions

**DOI:** 10.1186/s12917-023-03785-z

**Published:** 2023-10-20

**Authors:** Gerard Eduard Martin-Valls, Yanli Li, Hepzibar Clilverd, Jordi Soto, Martí Cortey, Enric Mateu

**Affiliations:** Departament de Sanitat i Anatomia Animals, Facultat de Veterinària, Travessera dels Turons S/N, Universitat Autònoma de Barcelona, 08193 Cerdanyola del Vallès, Spain

**Keywords:** Porcine reproductive and respiratory virus, Neutralizing antibodies, Broadly neutralizing antibodies, Vertical transmission

## Abstract

**Background:**

Vertical transmission is key for the maintenance of porcine reproductive and respiratory syndrome virus (PRRSV) infection. In vaccinated farms, vertical transmission can still occur despite sows having some level of immunity because of repeated vaccination or contact with the wild-type virus. The present study aimed to correlate the age of sows and the amplitude of neutralizing antibodies (Nab) (heterologous neutralization) with PRRSV-1 vertical transmission (VT). For this purpose, umbilical cords of 1,554 newborns (corresponding to 250 litters) were tested for PRRSV by RT-PCR in two PRRSV-unstable vaccinated farms. In parallel, the sows were bled after farrowing and the levels of antibodies were determined by ELISA and by the viral neutralization test against the vaccine virus, the virus circulating in the farm, and other unrelated contemporary PRRSV-1 strains. The relationship between the parity and the probability of delivering infected piglets and the presence of broadly Nabs examined.

**Results:**

The proportion of VT events in the two examined farms ranged from 18.9% to 23.0%. Young sows (parity 1–2) were 1.7 times more likely to have VT than older sows (*p* < 0.05). Despite higher ELISA S/P antibody ratios in younger sows (*p* < 0.05), NAb against the resident farm strain were at a similar level between sows delivering infected and healthy piglets regardless of age, mostly with low titers (2–3 log_2_). The titers of NAb against the vaccine virus were also low, and no correlations with VT were observed. When a panel of another 4 strains (1 isolated in the 1990s, and 3 contemporary strains) were used for the neutralization test, most sow sera were not capable of neutralizing the contemporary strains.

**Conclusions:**

Titers of NAb could not be correlated with the occurrence of PRRSV VT. The amplitude of NAb present in most vaccinated sows is limited with a considerable proportion unresponsive regarding NAb production.

**Supplementary Information:**

The online version contains supplementary material available at 10.1186/s12917-023-03785-z.

## Background

Porcine reproductive and respiratory syndrome (PRRS) was first described in the United States in 1987 [1] and now has become one of the costliest diseases in the pig industry [2, 3]. When PRRS virus (PRRSV) is introduced on a farm, the infection spreads rapidly among susceptible sows. If sows are infected in late gestation, transplacental infection may happen, resulting in abortion, mummified fetuses, or the birth of weak, congenitally infected piglets. These viraemic-born piglets will then bring the infection downstream to the nurseries and growing units. If the virus circulation in the breeding herd is maintained, the farm will become PRRSV endemic [4]. In endemic farms, vertical transmission (VT) is the main factor in perpetuating infection in nurseries. These farms, where the virus circulates in breeders and have viraemic piglets at weaning, are usually designated as unstable [5, 6]. Accordingly, new cycles of re-circulation along with an increase in VT events or reproductive problems are periodically observed if no control measures are implemented [7].

Current knowledge has not fully resolved what is the contribution of young and old sows in these endemic circulation cycles. Although neutralizing antibody (NAb) titers ≥ 1:16 have been demonstrated to protect against abortion in a homologous challenge model [8], the prediction of the efficacy of heterologous neutralizing antibodies is uncertain. As shown in several studies, the neutralizing capacities of the elicited PRRSV NAb could be determined by the specific strains to which the animal was exposed, the number of exposures, and other intrinsic factors of the host [8–10].

Vaccines are one of the main tools to control PRRSV infection. Live attenuated vaccines are preferred over inactivated ones for priming the gilts. Once immunized, periodic boosting is required. Although several vaccination programs are applied, blanket vaccination protocols (all sows at one time, every 3–4 months) are a popular strategy to maintain the immunity of breeders. Repeated administration of live attenuated vaccines is assumed to be safe when a good balance is achieved between viral replication to induce solid immunity and sufficient viral attenuation to prevent symptomatic disease. The objective of the present study is to determine whether the age of sows and the level and amplitude of NAb correlate with PRRSV-1 VT in vaccinated farms.

## Results

In Farm 1 (F1), 139 farrowings were followed, comprising 41 young sows (parities 1–2), 65 middle-aged sows (parities 3 to 6), and 33 old sows (parity ≥ 7). In this farm, VT of PRRSV occurred in 32 cases (23.0%; CI95% = 16.5—31.1%), of which 14 (43%) happened in young sows (*p* < 0.05). Of note, young sows only accounted for 30% of the sampling of this farm. In Farm 2 (F2), 111 farrowings were followed, including 51 young, 49 middle-aged, and 11 old sows. VT was detected in 21 farrowings (18.9%; CI_95%_ = 12.4—27.1%) with 15 (71%) happening in young sows (*p* < 0.001). The aggregated data from both farms examined in the generalized linear model (GLM) model (Table 1) showed that the age of sows had a significant influence on the occurrence of VT (*p* = 0.012). Overall, PRRSV VT occurred more likely, 1.7 times (CI_95%_ = 1.22—2.31), in young (parities 1–2) than in older sows.

**Table 1:** Generalized linear model results of the association of vertical transmission with regard to age

**FORMULA** > model <—glmer(VT ~ AGE + (1 | FARM), data = data, family = binomial, control = glmerControl(optimizer = "bobyqa"))					
**AIC**	**BIC**	**logLik**	**Deviance**	**df.resid**	
271.8	285.9	-131.9	263.8	245	
**Scaled residuals**					
**Min**	**1Q**	**Median**	**3Q**	**Max**	
-0.7190	-0.6454	-0.4666	-0.4188	2.3876	
**Random effects**					
**Groups**	**Name**	**Variance**	**Standard deviation**		
*Farm*	(intercept)	0.02988	0.1728		
	**Estimate**	**Standard Error**	**z value**	**Pr( >|z|)**	**Interpretation**
**Fixed effects**					
*Intercept*	-1.6344	0.2847	-5.741	9.44e-09	*p* < 0.001
*AgeYoung*	0.8648	0.3432	2.520 0.855	0.0117	*p* < 0.05
*AgeOld*	0.3806	0.4453		0.3927	non-significant
**Correlation of Fixed Effects**	(Intr)	AgeYoung			
*AgeYoung*^a^	-0.681				
*AgeOld*^b^	-0.473	0.388			

^a^Young sows were considered to be sows in parities 1 and 2

^b^Old sows were those of parity ≥ 7

**Fig. 1:**
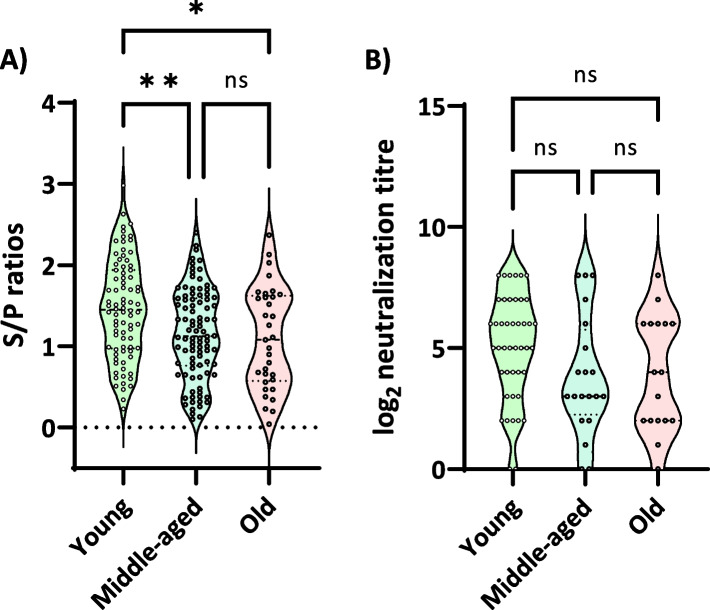
Antibody levels by age of the examined sows. The figure depicts the distribution of the results of serological analyses visualized by violin plots. **A** S/P ratios by ELISA (Idexx). All sows (F1, *n* = 139; F2, *n* = 111) were examined. **B** Log_2_ titer of neutralizing antibodies (NAb) against the vaccine strain. In this case, 80 sows were examined. Young = sows of parities 1–2, *n* = 37; Middle-aged = sows of parities 3–6, *n* = 20; Old = sows of parities ≥ 7, *n* = 17. * *p* < 0.05; **p < 0.01; n.s. = non-significant differences

Regarding the levels of antibodies measured by ELISA, no differences were observed between farms with regard to the same age group of sows (young, middle-aged, old) (data not shown). The global average S/P ratio was 1.4 ± 0.8 for sows that had VT (1.3 and 1.5 for F1 and F2, respectively), and 1.2 ± 0.6 for sows delivering healthy piglets (1.2 and 1.3 for F1 and F2, respectively). Interestingly, younger sows, despite the VT status, had significantly higher S/P values than older ones (1.4 ± 0.6 vs 1.1 ± 0.6, *p* < 0.05) (Fig. 1A).

Next, we examined whether the titer of NAb against the resident farm strain was correlated with the delivery of infected or healthy piglets on F1 (the same analysis was not performed for F2 because the resident farm strain could not be adapted to MARC-145). Twenty VT-sows (all sows for which enough serum was available for the viral neutralization test (VNT)) and 69 non-VT-sows were analyzed. The average neutralization titer was 2.3 ± 2.0 log_2_ for sows delivering healthy piglets versus 3.1 ± 2.3 log_2_ for sows delivering infected piglets (non-significant, Supplementary Fig. 1).

Then the relationship between the titers of PRRSV-specific NAb (vaccine strain Porcilis® PRRS) and the occurrence of VT was analyzed. The average titer of sows with occurrence of VT did not differ from those of sows delivering PRRSV-negative piglets (2.1 ± 0.5 vs 2.2 ± 0.4 log_2_ in F1 and 5.1 ± 2.5 and 4.0 ± 2.4 log_2_ in F2 for VT and non-VT sows, respectively). When taking both farms together, no differences were also observed between VT and non-VT sows. Younger sows showed a trend for higher neutralization titers compared to middle-aged or older sows (5.1 ± 2.2 vs 4.0 ± 2.5 vs 3.7 ± 2.0 log_2_, respectively, *p* = 0.07, Fig. 1B).

**Fig. 2:**
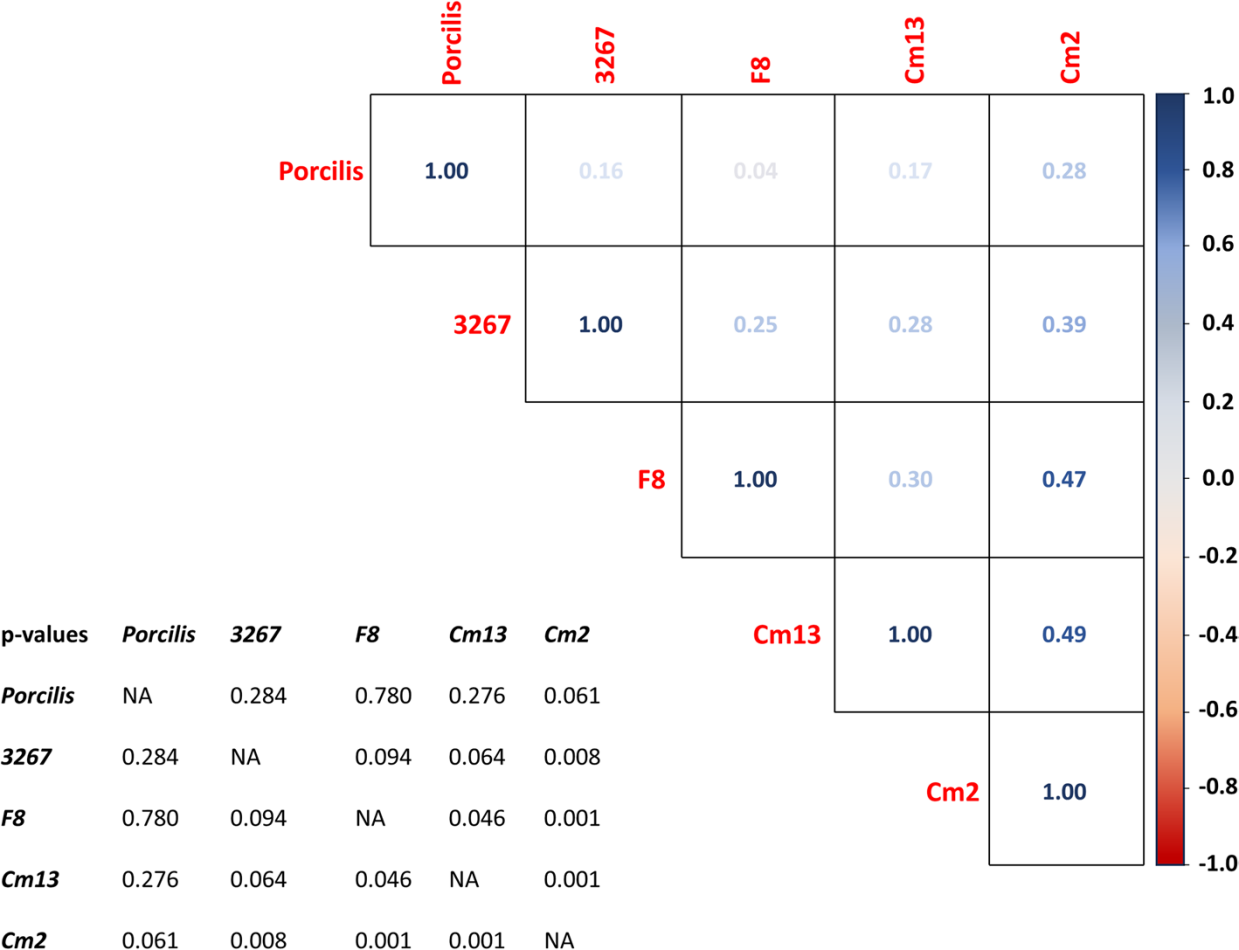
Correlation values (Spearman’s rho) for the viral neutralization tests using five strains. The figure shows rho values (within each square) for the correlation of neutralizing antibody (NAb) titers in sows (*n* = 51 with 24 young, 27 middle-aged and old sows) against 5 different strains: Porcilis, 3267, F8, Cm13 and Cm2. Asterisks indicate significant correlation values, detailed *p*-values for each comparison can be found in the table

Given differences observed in the antibody levels based on age, further analyses of antibody levels and the depth of neutralizing capacities of sera from sows of different ages was performed. Since no more serum of the abovementioned sows was available, these analyses were performed with sera from another group of 51 sows of F1 and F2 collected for routine monitoring purposes. The serum samples were analyzed in parallel by ELISA and the VNT against 5 PRRSV-1 strains (Table 2). Consistently, young sows displayed higher S/P values (data not shown). With regards to the neutralization against vaccine strain, the proportion of sows with neutralization titer ≥ 1:4 was 79.2% (19/24) and 85.2% (24/27) in young (parities 1–2) and older sows (parity ≥ 3), respectively (non-significant). The average titers were 4.30 ± 1.44 and 4.40 ± 1.40 log_2_, respectively (non-significant). Furthermore, the cross-neutralizing capability was evaluated against four heterologous PRRSV1 stains (3267, F8, Cn13, and Cw2). For 3267, 66.7% and 70.3% of young and adult sows respectively harbored NAb. For three contemporary strains (F8, Cn13, and Cw2), the proportion of positive sows was respectively 4.2%, 16.7%, and 16.7% in young sows, and 14.8%, 40.7%, and 29.6% in older sows. A trend towards signifcance was observed for the comparison of the proportions of positive sows in each group of age when strain Cn13 was used (*p* = 0.07). Mean titers ranged from 2.0 log_2_ for the F8 strain to 4.7 log_2_ for the Cn13 strain, with no significant differences between strains or between young and older sows. Only one serum from an adult sow was able to neutralize all five strains at titers higher than 1:4. Seven sera, all from adult sows (parity> 2), neutralized 4 strains (all but F8), 9 sera (6 young and 3 adult sows) neutralized 3 strains (Porcilis®, 3267 and one of the three contemporary strains), and 14 sera neutralized 2 strains (Porcilis and 3267, except in one case). The remaining 5 sera (2 young sows and 3 sows of parity ≥ 3) did not neutralize Porcilis® or any of the other strains. The correlation of NAb titers in sows against each of the five strains is shown in Fig. 2. Titers of neutralization against the vaccine strain were not significantly correlated to the titers against any other strain. In contrast, titers against strain Cm2 were significantly correlated (*p* < 0.05) with all strains but not Porcilis® although a significant trend was observed (*p* = 0.061).

**Table 2:** Nucleotide sequence identity matrix for the PRRSV-1 strains used for neutralization in the present study.

	**Resident F1**	**Cresa3267**	**MLV-DV**	**Cw2**	**Cn13**	**F8**
Resident F1	ID	0.8450	0.8433	0.8355	0.7871	0.7896
Cresa3267		ID	0.9487	0.8443	0.8327	0.8403
MLV-DV			ID	0.8424	0.8468	0.8565
Cw2				ID	0.7899	0.7934
Cn13					ID	0.7829
F8						ID

## Discussion

The results of the present study suggest that VT events occurred preferentially in young sows. The contribution of young sows to vertical and subsequent horizontal transmission has been reported for viral and bacterial diseases [11, 12]. However, in the case of PRRSV, the focus has been on the introduction of non-acclimated or non-vaccinated gilts. Usually, the role of young sows in the transmission of infectious agents is related to the lack of robust immunity against a given pathogen. In the present case, all sows had been vaccinated at least twice with an MLV before mating and since then they had received three vaccine doses per year. Almost all the examined sows had detectable antibodies by ELISA indicating that they had been in contact with the virus, either vaccine or wild type.

According to our results, four observations are worth mentioning. First, neither S/P ratios in ELISA nor titers of NAb against the resident farm strain increased with age. In addition, NAb against the vaccine virus were detected in just 75–80% of the sows regardless of age. Given that animals were vaccinated twice as gilts and then three times per year after the first service, it seems unlikely that the absence of NAb resulted from failed vaccination practices. The existence of unresponsive sows to vaccination in terms of developing antibodies was reported before [13, 14]. Bassaganya-Riera et al. [15] also showed that repeated immunizations with the same MLV did not significantly enhance antibody responses in sows. Our results would agree with that.

Second, despite NAb titers were not different between young and older sows, young sows were more likely to produce VT events. Vilalta et al. [16] reported that VT events may occur for several months since the initial outbreak, accumulating in the litters of young sows. Although no clear explanation can be proposed, our results suggest that young sows, even being vaccinated, might be more likely to get infected and develop viremia, allowing the virus to reach the placenta. Thus far, mechanisms involved in protection against PRRSV transplacental infection are largely unknown although recent publications suggest that T-cell immunity and IFN-γ responses are related [17, 18]. In human models like human cytomegalovirus, viral transmission to the fetus during pregnancy is also controlled by cell-mediated immunity [19].

The third finding is that most sows were only able to neutralize the vaccine virus or an old PRRSV-1 strain but not the contemporary ones, and titers against the resident farm strain were on average 3 log_2_ lower than those produced against the vaccine. It has been proposed that homologous NAb may protect sows against abortion if 4 log_2_ is reached [8]. Therefore, NAb in the sows of the present study were mostly able to neutralize the vaccine strain (or closely related ones) but probably not other heterologous strains.

Previous reports suggested that under farm conditions, broadly NAb (bNAb) were only developed in a small proportion of sows [20, 21]. Their development is assumed to depend on the infecting strain [9, 10] and probably also on repeated contact with various viral strains and the individual idiosyncrasy of the sows [19]. It has been proven that broadly reactive antibodies may confer protection against challenge with a heterologous PRRSV-1 strain [10]. Therefore, in endemic farms where sows are exposed to only the vaccine virus or the resident PRRSV strain, with limited introduction of other isolates, bNAb are less likely to develop in most sows.

Finally, the fourth finding is that sera of five sows did not show any neutralizing capability against the panel of five PRRSV-1 strains, including the vaccine strain. This suggests the existence of a proportion of sows that can be qualified as bad- or non-responders. As mentioned above, this observation has also been previously reported [13, 14]. Studies aiming at examining the role of unresponsive sows in PRRSV-endemic farms and identifying host genetic factors or immune contexts causing such a phenomenon would help to design a more effective vaccine.

Taken together, and in the context of the examined farms, the present study supports the notion that humoral responses of sows had very little predictive value with regard to determining the occurrence of VT incidence. This also opens the question of the protective value of colostral antibodies. Given the genetic diversities of pigs and antigenic variabilities of PRRSV strains, it cannot be categorically stated that the situation would be the same for all strains or farms. Broader studies examining more farms would be necessary to enlighten this point. Furthermore, the reasons why younger sows were at a higher risk of producing VT cases remain unclear. Cell-mediated immunity against PRRSV in the pregnant sow is an evident to-be-determined factor.

## Methods

### Farm selection and sampling

Two farrow-to-feeder PRRSV-1-positive unstable farms were selected for the present study. Farm 1 had a 300-sow stock yielding 24 deliveries every two weeks. Farm 2 was a 1,400-sow facility with 65 parturitions every week. Both farms were vaccinating gilts against PRRSV-1 with a modified live vaccine (MLV; Porcilis® PRRS, MSD, Spain) twice before the first service. Then, all sows received a recall blanket vaccination 3 times per year with the same vaccine. As previously examined, these farms had 20% of PRRSV-1-positive litters at weaning; accordingly, it was considered that PRRSV VT cases could be found in a similar proportion (20% ± 7.5%, 95% confidence). Five days before parturition, 139 (F1) and 111 (F2) serum samples were collected from sows by ear vein venepuncture. On the day of birth, at least 6 umbilical cords (UC) from piglets [22] of each serum-sampled sow were collected, in total *n* = 1,554 piglets. After sampling, the animals remained on the farm and followed the normal production cycle.

### Sample analysis

Umbilical cords were examined for the presence of PRRSV by RT-PCR as previously described [22]. Serum samples from all sows (F1, *n* = 139; F2, *n* = 111) were first and analyzed by a commercial ELISA (IDEXX PRRS X3 Ab Test, IDEXX, United States). Selected sera (based on the availability of the sample volume) were further analyzed by viral neutralization test (VNT) for two purposes: 1) to examine whether titers of Nab could be related to the delivery of healthy or infected piglets, and 2) to assess the amplitude of Nab in sows of different ages. For the first purpose, sera from 89 sows of F1 (20 sows delivering infected piglets and 69 delivering healthy piglets) were tested by VNT against the farm resident strain in F1 (Genbank Accession n° OQ440238). VNT was not performed for F2 because the virus circulating in the farm could not be adapted to MARC-145.

Next, sera from 40 VT-sows and 40 non-VT-sows (both farms) were analyzed using the MLV strain as the antigen.

Finally, sera from a new set of 51 sows (24 of 1–2 parities with 12 from F1 and 12 from F2; 27 ≥ 3 parities with 13 from F1 and 14 from F2) were evaluated against a panel of 5 PRRSV-1 strains to assess Nab amplitude in sows of different ages regardless of the VT status. The 5 strains were: Porcilis® PRRS (Genbank Accession n° MT311646), 3267 Genbank Accession n° JF276435, isolated in Spain in the 1990s and used in previous papers [23]), Cw2 (Genbank accession n° OQ440239), Cn13 (Genbank Accession n° OQ440241), and F8 (Genbank Accession n° OQ440242). The latter three strains were isolated in the same geographical area as F1 and F2 since 2018. Based on the whole genome sequence comparison, similarities between the four strains and the vaccine strain ranged from 83.1% to 93.3%, and from 79.5 to 85.7% between the four strains (Table 2). VNT was performed on MARC-145 cell monolayers as previously described [24] with minor modifications. The neutralization titer was determined by the reciprocal of the highest serum dilution that produced 100% inhibition of the cytopathic effect in two replica wells. Persons performing the neutralization tests did not know the status of each sow regarding VT or age.

### Statistical analysis

Firstly, sows were categorized as young (1–2 parturitions), middle-aged (3–6 parturitions), and old (≥ 7 parturitions). The number of PRRSV-1 VT litters was compared between the three groups of sows using a Chi-square test with Yates correction, and the relative risk per farm was calculated using Koopman's likelihood-based approximation. To evaluate the age of sows on PRRSV-1 VT incidence, a generalized linear mixed model in Rstudio was used considering ‘age’ as the fixed effect and ‘farm’ as a random effect. S/P ratios were compared between groups of different ages using a Kruskal–Wallis on Statsdirect 3.2.10. The same analysis was also performed for sows delivering PRRSV-1 positive or negative piglets. Neutralization titers were log2 normalized and were compared using an ANOVA test on Statsdirect 3.2.10. Additionally, the proportion of positives was compared between groups with different ages against different strains. Correlation of results (titers) obtained in the VNT for the 5 strains was performed by means of the Spearman rho in RStudio.

### Supplementary Information

**Additional file 1:** **Supplementary Figure 1. **Distribution of neutralizing antibody titres (resident strain, farm 1) in sows that delivered viremic or healthy piglets. n.s. = non-significant. 

## Data Availability

Virus sequences produced in the present study have been deposited in Genbank under accession numbers OQ440238-OQ440242. Data, sera and the viruses may be shared upon reasonable request.

## Abbreviations

ELISA: Enzyme-linked immunosorbent assay
bNAb: Broadly neutralizing antibodies
GLM: Generalized linear model
MLV: Modified live vaccine
NAb: Neutralizing antibodies
PRRS: Porcine reproductive and respiratory syndrome
PRRSV: Porcine reproductive and respiratory syndrome virus
UC: Umbilical cord
VNT: Viral neutralization test
VT: Vertical transmission

## References

[1] Keffaber KK (1989). Reproductive failure of unknown etiology. Am Assoc Swine Pract Newsl.

[2] Holtkamp DJ, Kliebenstein JB, Neumann EJ, Zimmerman JF, Rotto HF, Yoder TK, Wang C, Yeske P, Mowrer CL, Haley CA (2013). Assessment of the economic impact of porcine reproductive and respiratory syndrome virus on United States pork producers. J Swine Health Prod.

[3] Renken C, Nathues C, Swam H, Fiebig K, Weiss C, Eddicks M, Ritzmann M, Nathues H (2021). Application of an economic calculator to determine the cost of porcine reproductive and respiratory syndrome at farm-level in 21 pig herds in Germany. Porcine Health Manag.

[4] Pileri E, Mateu E (2016). Review on the transmission porcine reproductive and respiratory syndrome virus between pigs and farms and impact on vaccination. Vet Res.

[5] Holtkamp DJ, Polson DD, Torremorell M, Morrison B, Classen DM, Becon L, Henry S, Rodibaugh MT, Rowland RR, Snelson H, Straw B, Yeske P, Zimmerman J (2011). Terminology for classifying swine herds by porcine reproductive and respiratory syndrome virus status. J Swine Health Prod..

[6] Holtkamp D, Torremorell M, Corzo CA, Linhares DCL, Almeida MN, Yeske P, Polson DD, Becton L, Snelson H, Donovan T, Pittman J, Johnson C, Vilalta C, Silva GS, Sanhueza J (2021). Proposed modifications to porcine reproductive and respiratory syndrome virus herd classification. J Swine Health Prod.

[7] Nodelijk G, Nielen M, De Jong MC, Verheijden JH (2003). A review of porcine reproductive and respiratory syndrome virus in Dutch breeding herds: population dynamics and clinical relevance. Prev Vet Med.

[8] Osorio FA, Galeota JA, Nelson E, Brodersen B, Doster A, Wills R, Zuckermann F, Laegreid WW (2002). Passive transfer of virus-specific antibodies confers protection against reproductive failure induced by a virulent strain of porcine reproductive and respiratory syndrome virus and establishes sterilizing immunity. Virology.

[9] Martínez-Lobo FJ, Díez-Fuertes F, Simarro I, Castro JM, Prieto C (2011). Porcine Reproductive and Respiratory Syndrome Virus isolates differ in their susceptibility to neutralization. Vaccine.

[10] Martínez-Lobo FJ, Díez-Fuertes F, Simarro I, Castro JM, Prieto C (2021). The ability of porcine reproductive and respiratory syndrome virus isolates to induce broadly reactive neutralizing antibodies correlates with in vivo protection. Front Immunol.

[11] Calsamiglia M, Pijoan C (2000). Colonisation state and colostral immunity to Mycoplasma hyopneumoniae of different parity sows. Vet Rec.

[12] Eddicks M, Beuter B, Stuhldreier R, Nolte T, Reese S, Sutter G, Ritzmann M, Fux R (2019). Cross-sectional study on viraemia and shedding of porcine circovirus type 2 in a subclinically infected multiplier sow herd. Vet Rec.

[13] Díaz I, Genís-Jorquera B, Martín-Valls GE, Mateu E (2020). Using commercial ELISAs to assess humoral response in sows repeatedly vaccinated with modified live porcine reproductive and respiratory syndrome virus. Vet Rec.

[14] Fiers J, Tignon M, Cay AB, Simons X, Maes D (2022). Porcine Reproductive and Respiratory Syndrome virus (PRRSv): a cross-sectional study on ELISA seronegative, multivaccinated sows. Viruses.

[15] Bassaganya-Riera J, Thacker BJ, Yu S, Strait E, Wannemuehler MJ, Thacker EL (2004). Impact of immunizations with porcine reproductive and respiratory syndrome virus on lymphoproliferative recall responses of CD8+ T cells. Viral Immunol.

[16] Vilalta C, Sanhueza J, Alvarez J, Murray D, Torremorell M, Corzo C, Morrison R (2018). Use of processing fluids and serum samples to characterize porcine reproductive and respiratory syndrome virus dynamics in 3 day-old pigs. Vet Microbiol.

[17] Li Y, Diaz I, Martin-Valls G, Beyersdorf N, Mateu E (2023). Systemic CD4 cytotoxic T cells improve protection against PRRSV-1 transplacental infection. Front Immunol.

[18] Stas MR, Kreutzmann H, Stadler J, Sassu EL, Mair KH, Koch M, Knecht C, Stadler M, Dolezal M, Balka G, Zaruba M, Mötz M, Saalmüller A, Rümenapf T, Gerner W, Ladinig A (2022). Influence of PRRSV-1 vaccination and infection on mononuclear immune cells at the maternal-fetal interface. Front Immunol.

[19] Lilleri D, Gerna G (2016). Maternal immune correlates of protection from human cytomegalovirus transmission to the fetus after primary infection in pregnancy. Rev Med Virol.

[20] Robinson SR, Li J, Nelson EA, Murtaugh MP (2015). Broadly neutralizing antibodies against the rapidly evolving porcine reproductive and respiratory syndrome virus. Virus Res.

[21] Robinson SR, Rahe MC, Gray DK, Martins KV, Murtaugh MP (2018). Porcine reproductive and respiratory syndrome virus neutralizing antibodies provide in vivo cross-protection to PRRSV1 and PRRSV2 viral challenge. Virus Res.

[22] Martín-Valls GE, Hidalgo M, Cano E, Mateu E (2018). Testing of umbilical cords by real time PCR is suitable for assessing vertical transmission of porcine reproductive and respiratory syndrome virus under field conditions. Vet J.

[23] Díaz I, Gimeno M, Darwich L, Navarro N, Kuzemtseva L, López S, Galindo I, Segalés J, Martín M, Pujols J, Mateu E (2012). Characterization of homologous and heterologous adaptive immune responses in porcine reproductive and respiratory syndrome virus infection. Vet Res.

[24] Yoon KJ, Zimmerman JJ, Swenson SL, McGinley MJ, Eernisse KA, Brevik A, Rhinehart LL, Frey ML, Hill HT, Platt KB (1995). Characterization of the humoral immune response to porcine reproductive and respiratory syndrome (PRRS) virus infection. J Vet Diagn Invest.

