# Supplementary material for: Levels of neutralizing antibodies against resident farm strain or vaccine strain are not indicators of protection against PRRSV-1 vertical transmission under farm conditions
Source: BMC Vet Res. 2023 Oct 20;19:217. doi: 10.1186/s12917-023-03785-z (PMC10588270; doi:10.1186/s12917-023-03785-z)
Supplement: Supplementary file 1 — Additional file 1: Supplementary Figure 1. Distribution of neutralizing antibody titres (resident strain, farm 1) in sows that delivered viremic or healthy piglets. n.s. = non-significant. [file 12917_2023_3785_MOESM1_ESM.docx]

**Supplementary Figure 1. Distribution of neutralizing antibody titres (resident strain, farm 1) in sows that delivered viremic or healthy piglets**. n.s. = non-significant.
